# Unshrinking the Lung: Successful Improvement of Shrinking Lung Syndrome in Systemic Lupus Erythematosus Through Non-invasive Ventilation and Respiratory Rehabilitation

**DOI:** 10.7759/cureus.80400

**Published:** 2025-03-11

**Authors:** Pedro Fernandes, Ana Luis, João P Silva, António Reis

**Affiliations:** 1 Pulmonology, Unidade Local de Saúde (ULS) de Viseu Dão-Lafões, Viseu, PRT

**Keywords:** diaphragmatic dysfunction, non-invasive ventilation, respiratory rehabilitation, shrinking lung syndrome, systemic lupus erythematosus

## Abstract

Shrinking lung syndrome (SLS) is a rare pulmonary manifestation of systemic lupus erythematosus (SLE), characterized by progressive dyspnea and severe restrictive ventilatory impairment. The pathophysiology remains unclear, with proposed mechanisms involving diaphragmatic dysfunction, pleural inflammation, and neuromuscular impairment.

We report the case of a 38-year-old non-smoking woman with a history of SLE, presenting with worsening dyspnea and hypoxemic respiratory failure. Pulmonary function tests revealed a severe restrictive pattern, and nocturnal oximetry demonstrated significant desaturation. Imaging showed no interstitial lung disease. A structured respiratory rehabilitation program, combined with nocturnal non-invasive ventilation (NIV) using intelligent volume-assured pressure support, resulted in substantial clinical and functional improvement. Over six months, the patient experienced significant symptom relief, pulmonary function stabilization, and resolution of nocturnal desaturation.

This case highlights the potential role of NIV and pulmonary rehabilitation in improving respiratory function and quality of life in patients with SLS. Early diagnosis and a multidisciplinary approach are fundamental in optimizing patient outcomes. Further research is needed to establish standardized treatment protocols for SLS.

## Introduction

Systemic lupus erythematosus (SLE) is a chronic autoimmune disease characterized by multisystem involvement, including the pulmonary system. Pulmonary disease in SLE is common and encompasses pleural, parenchymal, and vascular abnormalities [1,2]. Among these, shrinking lung syndrome (SLS) is a rare yet significant manifestation, characterized by a progressive reduction in lung volumes, diaphragmatic dysfunction, and restrictive ventilatory impairment [3].

First described in the 1960s, SLS remains incompletely understood, with its pathophysiology likely involving a combination of diaphragmatic myopathy, pleural inflammation leading to reduced lung compliance, and neurological involvement affecting respiratory muscle function [3,4]. Clinically, patients usually present with progressive dyspnea, often in the absence of significant radiographic abnormalities, making diagnosis challenging. Pulmonary function tests typically reveal a severe restrictive defect with decreased forced vital capacity (FVC) but normal diffusing capacity for carbon monoxide (DLCO), distinguishing it from interstitial lung disease [5].

Despite its debilitating impact, standardized treatment guidelines for SLS are lacking. Corticosteroids and immunosuppressive agents remain mainstays of therapy, but their efficacy is variable [6,7]. Non-invasive ventilation (NIV) and structured respiratory rehabilitation have emerged as promising interventions, particularly in patients with significant diaphragmatic dysfunction [8,9]. Early recognition and a multidisciplinary approach are crucial to improving outcomes and preventing irreversible pulmonary decline.

This case highlights a unique presentation of SLS in a patient with SLE, emphasizing the successful management of severe restrictive impairment through nocturnal NIV and structured respiratory rehabilitation. These findings underscore the relevance of a time-sensitive and global approach to improve pulmonary function and, therefore, quality of life in patients with SLS.

## Case presentation

The authors present a case of a 38-year-old non-smoking woman with a history of SLE since her early 20s who presented with progressive exertional dyspnea (mMRC grade 3). She denied cough, sputum production, wheezing, chest pain, or peripheral edema. Her medical history was significant for a mechanical aortic valve replacement in November 2021, and she was receiving long-term immunosuppressive therapy with rituximab (1 g) along with bisoprolol (5 mg/day).

On initial examination, she had a body mass index of 28.2 kg/m². Pulmonary auscultation revealed globally reduced vesicular breath sounds without adventitious sounds. Arterial blood gas analysis demonstrated hypoxemic respiratory failure, with an SpO₂ of 49 mmHg, prompting the initiation of long-term oxygen therapy (3 L/min at rest).

**Figure 1:**
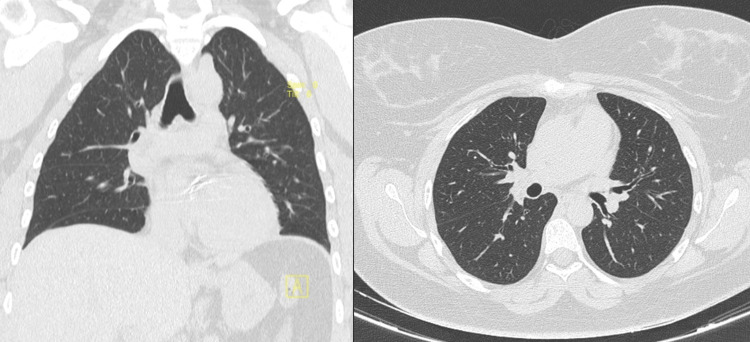
Chest CT scan showing no interstitial abnormalities, pleural effusions, or significant pulmonary pathology despite severe restrictive impairment CT: computed tomography

A chest CT scan revealed no interstitial abnormalities, pleural effusions, or other significant pulmonary pathology (Figure 1). Pulmonary function tests demonstrated severe restrictive impairment, with an FVC of 1.36 L (46% predicted), which further decreased by 3% in the supine position, a total lung capacity (TLC) of 2.44 L (59% predicted), and a DLCO of 89%. Additional assessments showed a peak cough flow of 270 L/min (normal >350 L/min), a maximum inspiratory pressure of -44 cmH₂O (normal >80 cmH_2_O), and a maximum expiratory pressure of 46 cmH₂O (normal >90 cmH_2_O). The six-minute walk test indicated exercise-induced desaturation, with SpO₂ dropping from 100% to 78% and a significant increase in dyspnea and fatigue scores.

A polysomnography study excluded sleep apnea but revealed substantial nocturnal desaturation, with a median SpO₂ of 86% and a T90 of 99%. Echocardiographic evaluation confirmed a well-functioning aortic prosthesis with mild transprosthetic regurgitation and preserved biventricular systolic function. There was no echocardiographic evidence of pulmonary hypertension.

**Figure 2:**
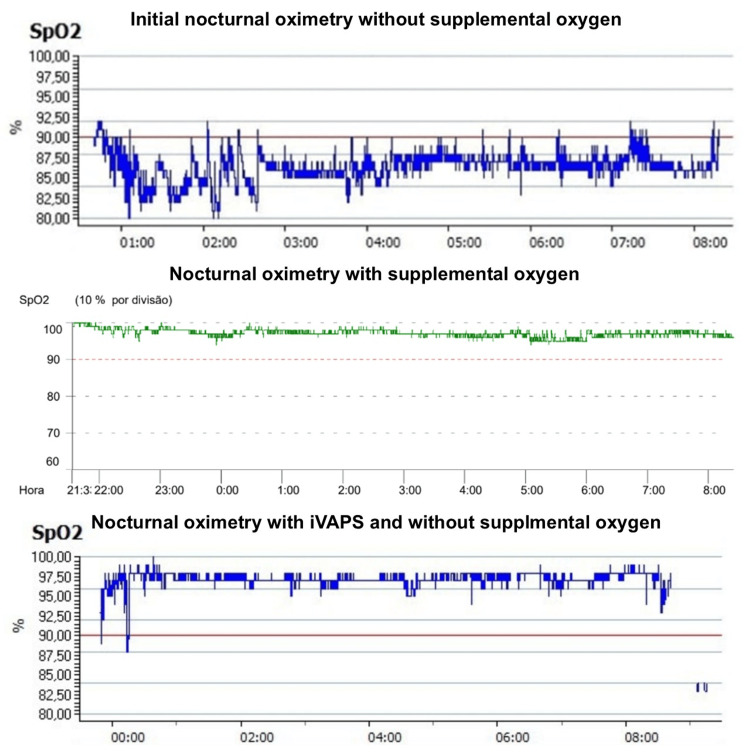
Nocturnal oximetry without supplemental oxygen, with supplemental oxygen, and with iVAPS without supplemental oxygen Initial nocturnal oximetry showing significant nocturnal desaturation​ with a median level of saturation of 86% and a T90 of 99%. Nocturnal oximetry with supplemental oxygen at 3 l/minute showing no desaturation, with a median level of saturation of 98%. Nocturnal oximetry after initiating NIV with iVAPS with resolution of the desaturation, with a median level of 98% and a T90 of 1% iVAPS: intelligent volume-assured pressure support, NIV: non-invasive ventilation, SpO_2_: partial pressure of oxygen

In August 2023, the patient was included in a structured respiratory rehabilitation program, incorporating nocturnal NIV using an intelligent volume-assured pressure support (iVAPS) mode. Initial NIV settings included an expiratory positive airway pressure of 6 cmH₂O and pressure support ranging from 5 to 10 cmH₂O. Within a few months, he could discontinue oxygen therapy at rest, requiring supplemental oxygen only during ambulation at 3 L/min (Figure 2).

Over six months, the patient demonstrated substantial improvements in dyspnea, vitality, and exercise tolerance. Compliance with NIV was excellent, with a median nightly usage exceeding six hours. Pulmonary function tests did not improve, with FVC 1.39 L (47% predicted), TLC 2.47 L (67% predicted), and DLCO 89%. Follow-up nocturnal oximetry confirmed the resolution of nocturnal desaturation, with a median level of 98% and a T90 of 1% (Figure 2). By early 2024, she had resumed daily activities with minimal reliance on supplemental oxygen.

## Discussion

This case underscores the importance of a multidisciplinary approach in managing SLS in patients with SLE. While SLS remains a rare and poorly understood manifestation of SLE, it significantly impacts pulmonary function and quality of life. The patient’s presentation of severe restrictive ventilatory impairment with profound desaturation highlights the need for early recognition and intervention [5,6].

The successful response to NIV and respiratory rehabilitation in this case aligns with emerging evidence suggesting that NIV can be beneficial in patients with diaphragmatic dysfunction [10]. iVAPS is an advanced mode of NIV designed to automatically adjust pressure support to maintain a target alveolar ventilation. It is primarily used for patients with chronic respiratory insufficiency, such as neuromuscular diseases or chronic obstructive pulmonary disease (COPD). iVAPS continuously adapts to the patient's changing needs by monitoring their breathing patterns and adjusting support to ensure stable ventilation. Although corticosteroids and immunosuppressive therapies are commonly employed in SLS, their effectiveness remains inconsistent and is related to important adverse effects [11-13], emphasizing the need for adjunctive strategies such as NIV. Using iVAPS mode in this patient ensured individualized ventilatory support, optimizing respiratory mechanics and gas exchange [3,5].

Additionally, pulmonary rehabilitation played a crucial role in improving exercise tolerance and dyspnea, reinforcing the value of structured physiotherapy programs in SLS management [14,15]. The stability of pulmonary function tests and the resolution of nocturnal desaturation further suggest that NIV and rehabilitation contribute to disease stabilization and functional recovery.

Despite the positive outcomes observed in this case, long-term follow-up remains essential to monitor disease progression and ventilatory function [16]. Further studies are needed to establish standardized treatment protocols and explore the role of NIV in modifying disease trajectory in SLS patients.

This case highlights the potential of early, individualized intervention in mitigating respiratory decline associated with SLS, emphasizing the necessity of ongoing research and clinical awareness to optimize patient outcomes [17].

## Conclusions

This case report demonstrates the potential benefits of NIV and structured respiratory rehabilitation in managing SLS in SLE. The patient’s significant clinical improvement following the introduction of nocturnal iVAPS underscores the importance of early, individualized intervention in mitigating disease progression and enhancing quality of life.

The findings suggest that a multidisciplinary approach, incorporating NIV and pulmonary rehabilitation, can lead to meaningful stabilization in patients with SLS. Further research is warranted to establish standardized treatment guidelines and determine the long-term efficacy of these interventions in managing this rare but debilitating condition.
